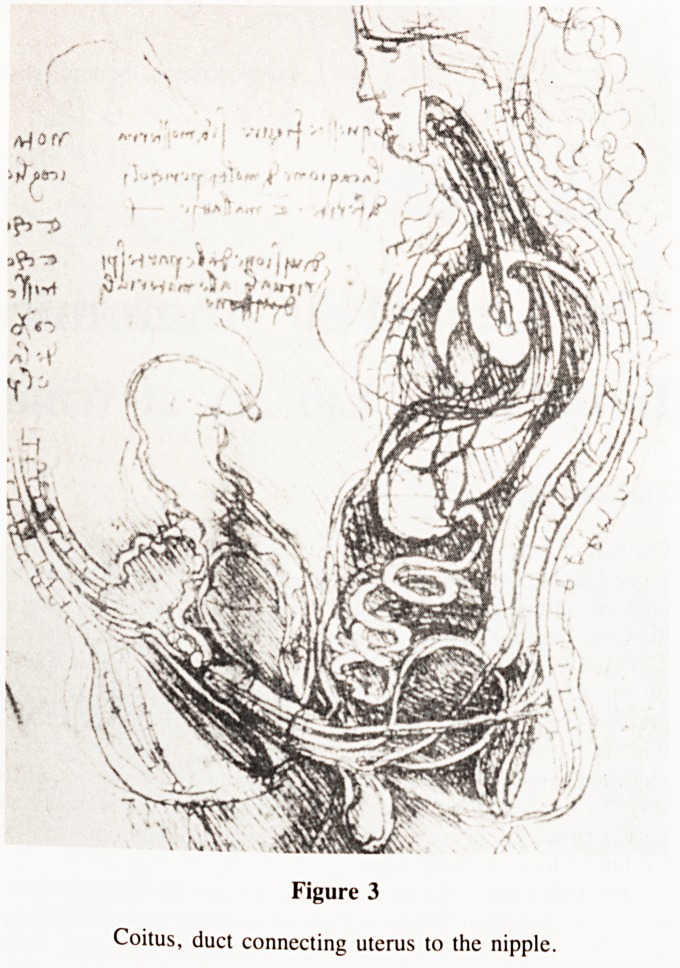# The Leonardo Anatomical Drawings

**Published:** 1991-12

**Authors:** Michael Wilson


**Fig. 2.**

Consequences of the dorsal position. (Dunn, P.M. *Lancet 1,* **790,** 1976).

### The Leonardo Anatomical Drawings

### In the Royal Library at Windsor Castle

**Michael Wilson, ChM, FRCS**

On July 22nd 1987 a small party of members of the Society visited Windsor Castle by appointment to see a selection of the anatomical drawings of Leonardo da Vinci. There are several hundred of these in folios in the Royal Collection and these are all listed in Volume 3 of 'The drawings of Leonardo da Vinci' by Sir Kenneth Clark. Before going we were asked to select 20 drawings from the list and when we arrived these were laid out for us on display tables. The drawings themselves were mounted between boards with transparent plastic windows, each sheet of paper having drawings on both sides. We were given gloves to wear and were allowed to handle the mounts so that we could look at both sides.

Leonardo's drawings were made in the late fifteenth and early sixteenth centuries. Works on human anatomy before this time were rare and, because printing had not been invented, were in manuscript, subject to the errors of copyists and therefore contained only basic and also usually inaccurate drawings. What we saw in these drawings was probably the first accurate depiction of human anatomy ever made. When one also considered that Leonardo was primarily an artist with little medical background and imagined the difficulty of dissection under conditions of secrecy, in the presence of putrefaction, without proper instruments, illumination or even perhaps running water one could only look at these drawings with awe and amazement, enhanced by the strange and beautiful mirror writing which surrounded them. Leornardo is reported to have said that he had dissected 30 bodies.

The drawings were nearly all on white paper in pencil inked with sepia. One's first impression was their accuracy and detail. They seemed clearer and more beautiful than the reproductions that one has been accustomed to see in books, probably because they were small and in many instance had been photographically enlarged for reproduction. I took especial interest in the drawing of the brachial plexus (fig. 1) because it is a complicated piece of anatomy which is nevertheless clearly defined and not subject to much variation. I checked it with my own atlas of anatomy and found it accurate and complete in all respects from the cervical and thoracic nerves of origin to the upper, middle and lower trunks, the anterior and posterior divisions giving rise to the medial, lateral and posterior cords leading ultimately to the median, ulnar, musculo-cutaneous and radial nerves. The sections of the skull showed accurately the frontal and maxillary sinuses not previously described. The asymmetric testicular veins ending differently on the two sides were accurately shown (Fig. 2). So many of the drawings seemed perfect that it was particularly fascinating to find that sometimes Leonardo had gone wrong and one can guess that he depicted what he believed ought to have existed if only he could have seen it. For example according to the 'humoral theory' which held sway at that time it was believed that 'black bile' was manufactured in the spleen and transferred to the liver and so ducts between the spleen and the liver are shown. (Fig. 2). None of the drawings of the liver show any biliary apparatus and Leonardo apparently was unaware of the existence of the pancreas. When it came to the heart and lungs one knew that it wasn't until 1650 that Harvey first unravelled the mystery of the circulation of the blood.

**Figure 1**

The Brachial Plexus.

**Figure 2**

Vascular connections of abdominal viscera. Vessels passing directly from spleen to liver may have been for transmission of Black Bile, according to 'humoral theory'.

**Figure 3**

Coitus, duct connecting uterus to the nipple.

Leonardo thought that air from the lung entered the heart and so one was not surprised to find some confusion about connections. Another fanciful drawing showed a duct connecting the uterus to the nipple (Fig. 3).

Leonardo's anatomical drawings were not generally known until 1898 when they were published in facsimile. How a large collection of Leonardo's drawings including the anatomical ones found their way into the Royal Collection is apparently unknown. Their first recorded appearance was when they were shown at Kensington Palace by Queen Mary in 1690. William Hunter knew about them and announced his intention of publishing them, but died in 1783 before he could do so. The first published anatomical atlas of Vesalius came out in 1543, some years after the invention of the printing press.

This was a memorable visit and we felt very privileged. Our grateful thanks were conveyed to Miss Henrietta McBurney, the deputy curator of the print room who laid out the exhibition and received us with great kindness.

## Figures and Tables

**Figure 1 f1:**
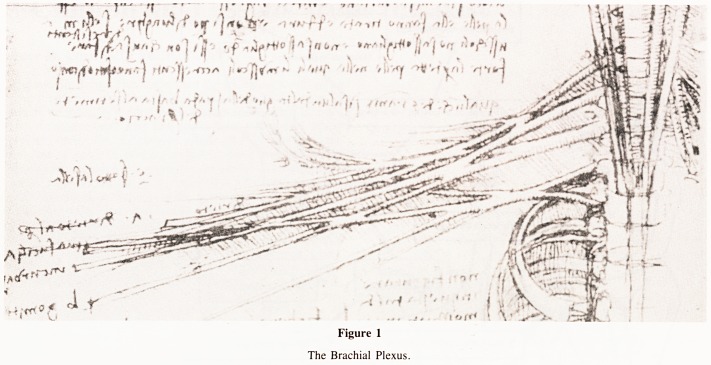


**Figure 2 f2:**
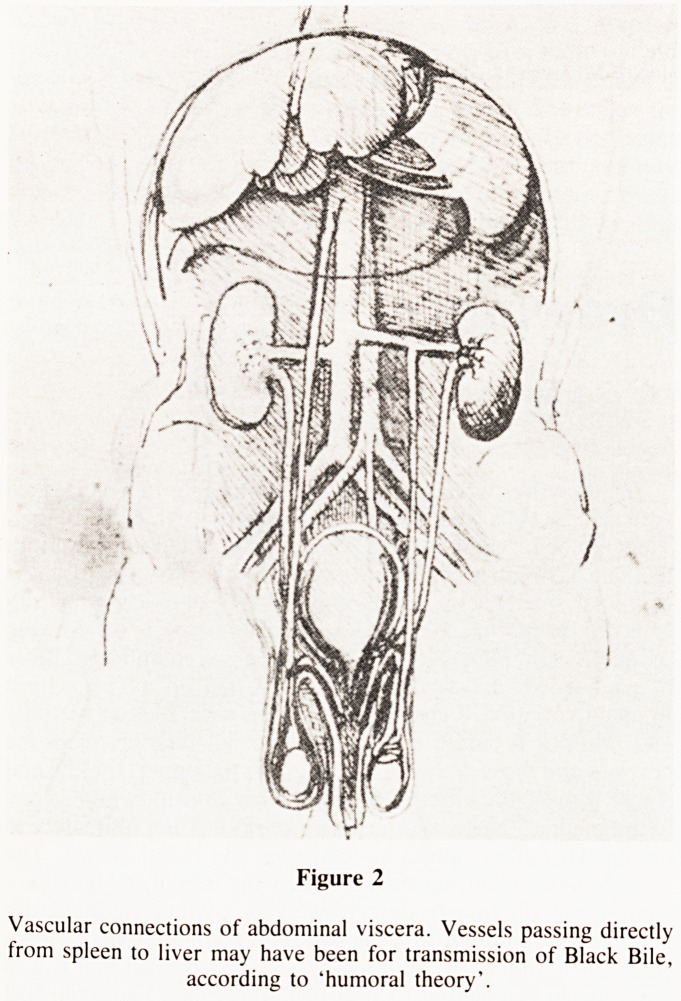


**Figure 3 f3:**